# Towards Fault-Aware Image Captioning: A Review on Integrating Facial Expression Recognition (FER) and Object Detection

**DOI:** 10.3390/s25195992

**Published:** 2025-09-28

**Authors:** Abdul Saboor Khan, Muhammad Jamshed Abbass, Abdul Haseeb Khan

**Affiliations:** 1 Department of Electrical Engineering and Information Technology, Otto-von-Guericke University, 39106 Magdeburg, Germany; 2 Faculty of Electrical Engineering, Wrocław University of Science and Technology, 27 Wybrzeze Stanisława Wyspianskiego St., 50-370 Wrocław, Poland; 3 Department of Computer Science, University of Kaiserslautern-Landau (Rheinland-Pfälzische Technische Universität Kaiserslautern-Landau), 67653 Kaiserslautern, Germany; khana@rptu.de

**Keywords:** image captioning, computer vision, deep learning, natural language processing, facial expression recognition, object detection, fault-aware systems, Prognostics and Health Management (PHM), Industry 4.0

## Abstract

The term “image captioning” refers to the process of converting an image into text through computer vision and natural language processing algorithms. Image captioning is still considered an open-ended topic despite the fact that visual data, most of which pertains to images, is readily available in today’s world. This is despite the fact that recent developments in computer vision, such as Vision Transformers (ViT) and language models using BERT and GPT, have opened up new possibilities for the field. The purpose of this review paper is to provide an overview of the present status of the field, with a specific emphasis on the use of facial expression recognition and object detection for the purpose of image captioning, particularly in the context of fault-aware systems and Prognostics and Health Management (PHM) applications within Industry 4.0 environments. However, to the best of our knowledge, no review study has focused on the significance of facial expressions in relation to image captioning, especially in industrial settings where operator facial expressions can provide valuable insights for fault detection and system health monitoring. This is something that has been overlooked in the existing body of research on image captioning, which is the primary reason why this study was conducted. During this paper, we will talk about the most important approaches and procedures that have been utilized for this task, including fault-aware methodologies that leverage visual data for PHM in smart manufacturing contexts, and we will highlight the advantages and disadvantages of each strategy. The purpose of this review is to present a comprehensive assessment of the current state of the field and to recommend topics for future research that will lead to machine-translated captions that are more detailed and accurate, particularly for Industry 4.0 applications where visual monitoring plays a crucial role in system diagnostics and maintenance.

## 1. Introduction

**Figure 1:**
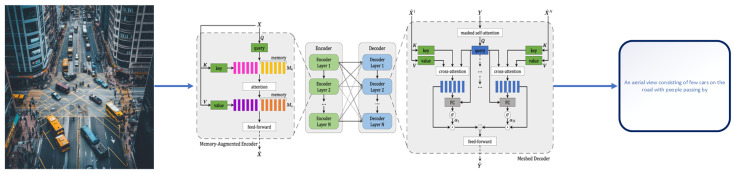
Typical application of image captioning systems.

Image captioning, a multidisciplinary field, involves producing machine-generated descriptions for images through the integration of computer vision, natural language processing, and deep learning techniques. Humans can easily evaluate images and generate textual summaries, whereas machines struggle with understanding contextual subtleties, recognizing objects, and detecting facial emotions. This field of research is gaining traction due to progress in deep learning, more data availability, and improved GPU performance. It highlights how textual summaries can be automatically generated from photographs without manual effort. For any input image I, the objective is to produce a caption C that precisely conveys the visual information contained in the image. Image captioning significantly improves machines’ ability to process multimodal data and is utilized across various domains, such as image processing and retrieval [1,2,3] human–machine collaboration [4], intelligent educational tools [5], and assistive technologies for the visually impaired [6], as well as advancements in social media platforms and surveillance systems, as depicted in Figure 1. Beyond these domains, a critical and emerging application lies within Industry 4.0, specifically in Prognostics and Health Management (PHM). In this high-stakes environment, a system’s ability to automatically interpret and describe a complex factory scene—identifying both equipment status and human factors—can provide invaluable, real-time insights for fault detection and operational safety. These systems generally obtain picture features through retrieval-based methods, reliant on manual feature engineering, or through deep learning approaches that independently extract prominent qualities using convolutional neural networks or advanced Vision Transformers (ViTs). Concurrently, the associated captions are produced utilizing recurrent models such as LSTMs or, more recently, sophisticated transformer architectures including BERT, GPT, or analogous decoders.

**Figure 2:**
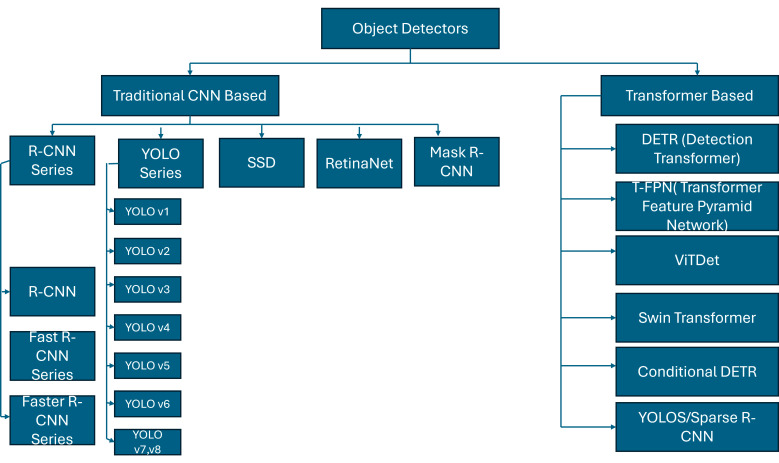
Taxonomy of convolutional neural network (CNN) and Transformer-based object detectors. Traditional CNN-based methods include R-CNN series [9,10,11], YOLO series (v1-v7) [12,13,14,15,16,17,18,19], SSD [20], RetinaNet [21], and Mask R-CNN [22]. Transformer-based approaches comprise DETR [23], T-FPN [24], ViTDet [25], Swin Transformer [26], Conditional DETR [27], YOLOS [28], and Sparse R-CNN [29].

One computer vision task is facial expression recognition (FER), which detects and categorizes human facial expressions based on their emotional content. The goal is to identify emotions like anger, fear, surprise, sadness, and happiness from a person’s facial features like eyebrows, eyes, and mouth in real-time due to the recent success of deep learning technologies in numerous sectors and the fact that facial expression recognition (FER) is moving from controlled laboratory settings to demanding real-world scenarios, as well as deep neural networks being increasingly employed to build discriminatory representations of automatic FER. The two main issues with the most recent deep FER systems are (1) insufficient training data and (2) expression fluctuations unrelated to contextual factors like lighting, head position, and identification biases [7]. In order to fully comprehend an image, object detection is used to identify and categorize specific items within it. Bounding boxes are created around observed items by object detection algorithms. In an industrial PHM context, these two technologies are not merely supplementary but synergistic. For instance, object detection can identify a physical anomaly like ’smoke’ from a ’motor’, while facial expression recognition can simultaneously detect an operator’s ’alarmed expression’. An integrated captioning system that understands both cues can generate a far more urgent and actionable description than a simple sensor alert ever could. Each bounding box is connected with a predicted class label and confidence score [8]. Figure 2 provides a high-level summary of the object detectors that are currently accessible.

**Figure 3:**
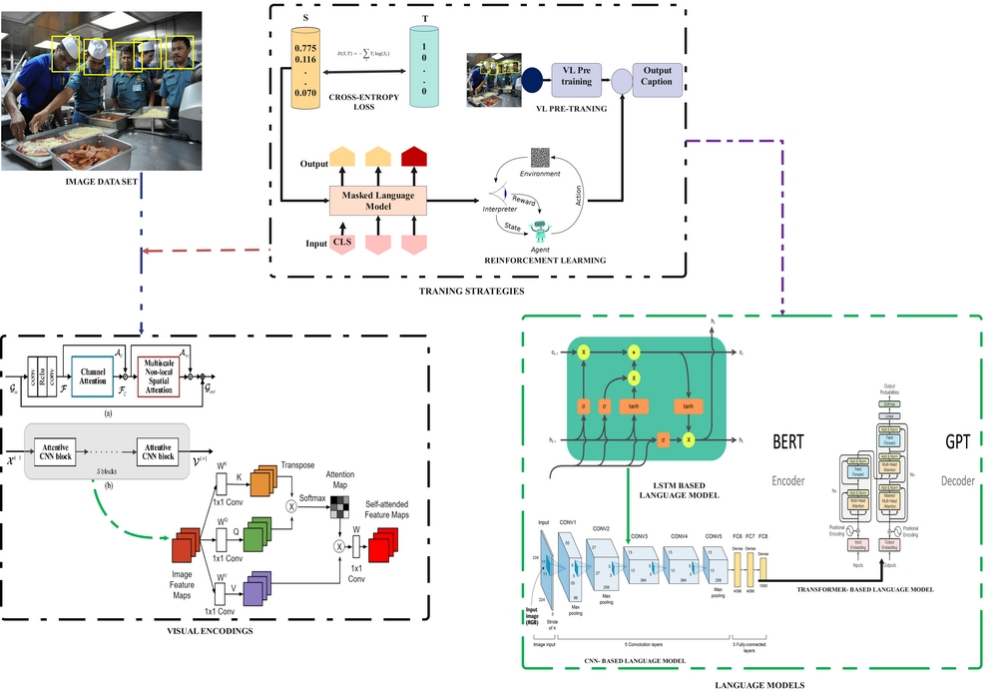
Overview of deep learning-based image captioning system [32].

Image captioning researchers realize that face sentiments prove beneficial for more accurate machine-translated captions and can lead to better results by adding emotions in the captions [30]. Deep learning models frequently use prior trained CNNs to generate high-level features from the final layers of a convolutional neural network. It provides a better grasp of the objects and their relationships in the image. Recently, substantial work has concentrated on leveraging attributes of the object for image captioning. Popular models, such as YOLOv3 [14], YOLOv4 [15], and YOLO9000 [13], noted based on their efficiency, precision, and near-real-time capabilities, have been used. Object features usually include object tags, which carry bounding box details, the predicted class, and the confidence rate. This study investigates the hypothesis that employing selected attributes can improve the quality of image captioning, whilst using all of the object aspects can assist with reproducing the human visual perception of situations more accurately. Although facial expression recognition is commonly conducted in uncontrolled outdoor settings, extracting emotions from static images under varying lighting conditions is complicated. In the last five years, the image captioning community has mainly focused on deep learning techniques as shown in Figure 3. Deep learning algorithms have demonstrated remarkable capabilities in addressing the complexities and challenges of image captioning. So far, no existing literature review article has specifically addressed the need to capture human sentiments and take advantage of object detection to localize the objects in an image. In spite of the fact that a few studies recently looked into the use of facial expressions for image captioning [30], and others have attempted the use of YOLOv4 [31], there is a noticeable gap in the research literature linked to the field of study in question. Presented here is a review article that focuses primarily on image captioning algorithms that combine facial expression recognition (FER) and object detection. This is shown in order to provide a concise summary of the existing body of expertise. Novel opportunities for making emotionally intelligent, context-rich captions arise when FER [30] and object detection [31] are combined with image captioning. This combination can improve the quality of machine-generated descriptions while giving them a greater depth of emotions, making them more similar to human understanding. While there have been significant developments in integrating object features and deep learning models for image captioning, there needs to be more research on how these technologies might be coupled to improve the accuracy of captions, allowing them to capture more complicated emotions and complex interactions between objects.

This review seeks to bridge this gap by providing an extensive review of image captioning algorithms that integrate FER with the detection of objects. We investigate this integration’s technological foundations, applications, problems, and future possibilities. The succeeding sections can be organized as follows: Section 2 provides a background and literature review, Section 3 discusses technological foundations in facial expression recognition, Section 4 examines object detection, Section 5 discusses the datasets, Section 6 examines the incorporation of FER and object detection, Section 7 examines the current trends and future directions, and Section 8 concludes the paper.

## 2. Background and Literature Review on Image Captioning for Fault-Aware PHM

### 2.1. Development of Image Captioning

Image captioning is a prominent field of study in Artificial Intelligence (AI) that focuses on comprehending images and generating a descriptive language for them, with growing relevance to fault-aware systems in Prognostics and Health Management (PHM) within Industry 4.0. In industrial settings, this involves not only detecting objects and relationships but also interpreting operator facial expressions to predict system faults, such as equipment malfunctions signaled by human stress indicators. Image understanding necessitates the ability to detect and identify objects. Additionally, it must possess the capability to comprehend the type or location of a scene, as well as the properties of objects and their interactions. To produce coherent sentences, one must possess a comprehensive grasp of both the syntax and semantics of the language [33], especially when captions need to alert maintenance teams to potential failures. The ability to comprehend an image largely hinges on the extraction of image features, which can be obtained through traditional machine learning techniques or deep machine learning techniques. In PHM applications, these features enable fault-aware captioning by linking visual cues (e.g., worn machinery parts or frustrated operators) to predictive diagnostics. These features can be obtained through two primary approaches: traditional machine learning techniques and deep machine learning techniques. A number of features, including Local Binary Patterns (LBPs) [34], Scale-Invariant Feature Transform (SIFT) [35], and the Histogram of Oriented Gradients (HOG) [36], as well as combinations of these, are often employed in traditional machine learning. These methods involve the extraction of data from input. The next step is to feed them into a classifier, like Support Vector Machines (SVMs) [37] so that the object may be classified. It is not possible to extract features from a big and varied dataset using handcrafted features because these characteristics are task specific. Furthermore, data from the actual world, such as pictures and videos, are complicated and might have various semantic meanings. Image captioning began as a bold attempt to translate visuals into words. Simple algorithms generated written descriptions from fundamental image attributes in the early days, laying the framework for more complicated computer vision–natural language processing interactions. For instance, in Industry 4.0, HOG might detect operator postures indicating fatigue, but its limitations in handling lighting variations in factory environments highlight the need for deep learning alternatives.

Deep machine learning strategies, such as convolutional neural networks (CNNs) [38], automatically learn features from training data and are better suited for complex industrial visuals like production line anomalies. CNNs have been pivotal in image classification [38,39,40,41,42,43,44] and object detection [10,11,22], forming the backbone of fault-aware systems where visual data informs PHM. For example, in [45], neural networks anticipated words based on images, a concept adaptable to generating captions like “Operator showing signs of frustration near overheating conveyor belt” for early fault intervention.

This section provides an overview of prominent image captioning systems (Figure 3), from visual encoding to language models, emphasizing their adaptation for PHM in Industry 4.0.

#### 2.1.1. Convolutional Neural Networks (CNNs)

Deep learning designs that are particularly useful for computer vision problems include convolutional neural networks (CNNs), which are among the most effective and frequently accepted architectures in the field. The idea was initially presented by Fukushima in his foundational work on the “Neocognitron” [46], which was motivated by the hierarchical receptive field model of the visual cortex that was proposed by Hubel and Wiesel. Following that, Waibel et al. [47] made advancements in CNNs by implementing backpropagation for phoneme recognition and providing shared weights for temporal receptive fields. Subsequently, LeCun and his colleagues [48] built a CNN architecture that was specifically designed for document recognition. This achievement represents a significant milestone in the realm of practical applications.

There are normally three basic types of layers that are included in CNNs. These layers are pooling, convolutional, and nonlinear. By employing kernels, also known as filters, the convolutional layers are able to extract spatial characteristics from the input data. On the other hand, the nonlinear layers make use of activation functions in order to capture complex nonlinear interactions. By summarizing local neighborhoods with statistical measures such as maximum or average values, pooling layers, on the other hand, downsample feature maps. This is accomplished by utilizing statistical measurements. Each layer is locally connected, which means that each unit processes information from a small, limited portion (receptive field) of the layer that came before it. CNNs build hierarchical feature representations by stacking many layers, which enables higher layers to capture increasingly abstract patterns over broader receptive fields. This is accomplished by stacking numerous layers.

As a result of weights being shared across receptive fields within the same layer, convolutional neural networks (CNNs) require a substantially lower number of parameters as compared to fully connected neural networks. The vast majority of contemporary CNN models are pre-trained on large-scale datasets such as ImageNet. Image captioning research frequently makes use of these pre-trained networks, extracting features from the final convolutional layers. In the literature, there are numerous CNN architectures that are generally recognized. Table 1 provides an overview of these models.

Most of the image captioning models are based on an encoder–decoder framework, with the encoder side dealing with Computer Vision Advancements, while the decoder starts with LSTM and GRU as well as the recent advancements in Transformers related to NLP.Visual encoding refers to the process of representing information or data using visual elements such as shapes, colors, and patterns. The primary obstacle in an image captioning process is to deliver a proficient depiction of the visual content. The existing methods for visual encoding can be categorized as non-attentive, attention over grid, and attention over visual regions. The taxonomy is visually depicted in Figure 3.

**Figure 4:**
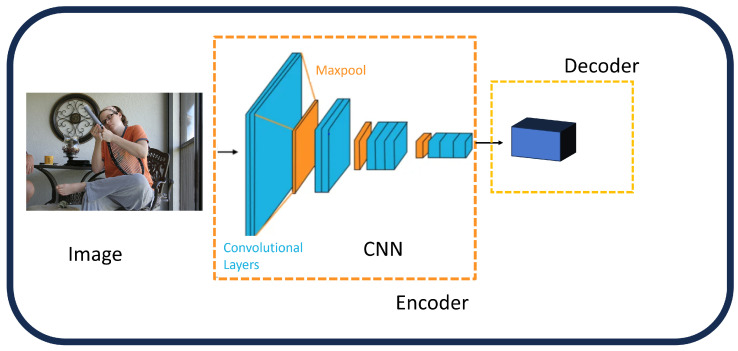
Global CNN features [32].

**Figure 5:**
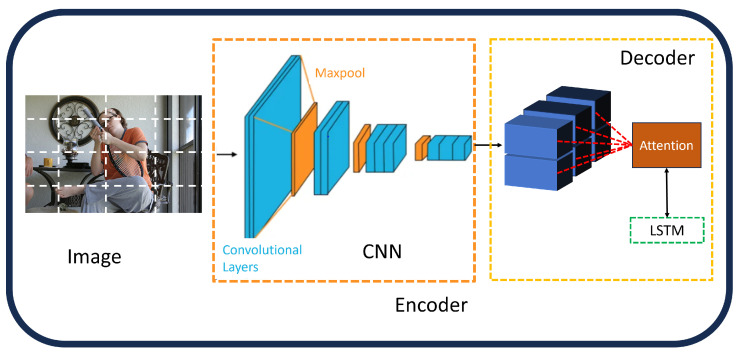
Grid CNN features [32].

- **Non-Attentive CNN Features:** The performance of models that handle visual inputs, such as those used for picture captioning, has significantly increased with the introduction of convolutional neural networks (CNNs). A straightforward approach involves extracting high-level image features from one of the final layers of a CNN, which then serve as conditioning signals for the language model (Figure 4). The output from GoogleNet [43] was used as the language model’s initial hidden state in the groundbreaking “Show and Tell” work [61], which was the first to employ this tactic. Global features obtained from AlexNet [40] were then used similarly by Karpathy et al. [62]. Furthermore, global features from the VGG network [42] were integrated into the language model at each time step by Mao et al. [63] and Donahue et al. [64]. Many image captioning architectures later adopted these global CNN properties as a key element [65,66,67,68,69,70,71,72]. For example, the FC model, which was proposed by Rennie et al. [73], encodes images using ResNet101 [44] while maintaining their original spatial dimensions. By using high-level semantic features or tags, which are represented as probability distributions over frequently occurring terms in the training captions, other approaches [74,75] improved the procedure. Global CNN features’ primary benefit is their compactness and simplicity, which allow for effective information extraction and contextual representation of the full image. The creation of accurate and fine-grained descriptions may be hampered by this method’s drawbacks, which include the excessive compression of visual features and a lack of granularity. In fault-aware image captioning for Industry 4.0, these non-attentive CNN features offer a compact baseline for rapid fault detection in manufacturing environments, such as identifying initial anomalies in machinery. However, their granularity limitations highlight the need for integration with FER and object detection to generate detailed captions that support PHM by incorporating operator expressions for enhanced system diagnostics.
- **Attention Over Grid:** In response to problems with global representations, some methods have improved visual encoding by making it more granular [73,76,77]. In order to include spatial structure directly into the language model, Dai et al. [78] used 2D activation maps instead of 1D global feature vectors. Instead, many in the image captioning community have been inspired by machine translation and have used the mechanism based on additive attention (Figure 5). This has given image captioning systems the ability to encode visual elements that change over time, which allows for more customization and finer definitions. The concept of additive attention can be understood as a form of weighted averaging, which was first introduced in a sequence alignment model by Bahdanau et al. [79], using a one-hidden layer feed-forward network in order to determine the score for attention alignment:
  (1) $$f_{att}\left(h_{i},s_{j}\right)=\;v_{a}^{T}tanh\left(W_{a}\left[h_{i};s_{j}\right]\right)$$
  where $v_{a}$ and $W_{a}$ are the learned attention parameters. Here, h and s are the encoder and decoder hidden states, respectively. This function is an alignment score function.
  An innovative method that utilizes additive attention on the convolutional layer’s spatial grid was presented by Xu et al. [76]. Using this technique, the model may zero in on specific areas of the grid by selecting appropriate feature subsets for each word in the output string. First, activations are obtained from the last convolutional layer of a VGG architecture [42]. Then, weights are assigned to particular grid points using additive attention, representing their relevance in predicting the next word.

In fault-aware image captioning for Industry 4.0, attention over grid mechanisms improve diagnostic accuracy by dynamically weighting relevant spatial features in industrial scenes, such as machinery faults. This integration with FER and object detection enables detailed captions that incorporate operator expressions, facilitating real-time PHM for predictive maintenance and system health monitoring.

**Figure 6:**
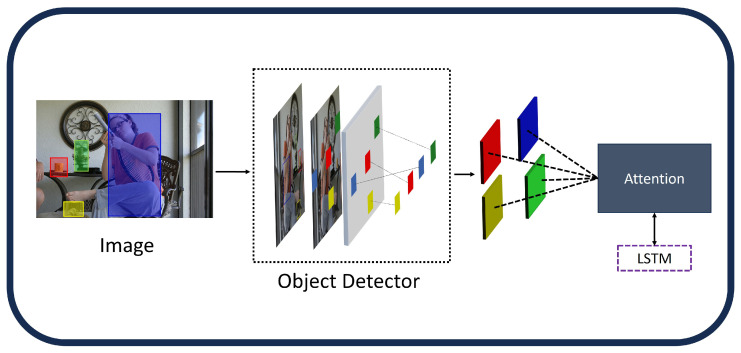
Attention over regions [32].

- **Attention Over Visual Regions:** Neuroscience indicates that the brain combines top–down cognitive processes with bottom–up visual signals to account for saliency. The top–down pathway utilizes prior knowledge and inductive bias to predict sensory inputs, whereas the bottom–up pathway adjusts these predictions according to actual visual stimuli. Top–down additive attention functions based on this principle. This method involves the language model forecasting the subsequent word by referencing a feature grid characterized by image-independent geometry, effectively integrating signals from both directions. Anderson et al. [80] present a bottom–up mechanism facilitated by an object detector that suggests visual regions, in contrast to conventional saliency-based approaches [81]. A top–down module is employed to assign weights to these regions for the purpose of word prediction (Figure 6). Faster R-CNN [11] is utilized for object detection, producing pooled feature vectors for each region proposal. The pre-training strategy employs an auxiliary loss to predict object and attribute classes using the Visual Genome dataset [82]. This allows the model to capture a comprehensive array of detections, encompassing salient objects and contextual regions, while developing robust feature representations.
  Image-area features have traditionally been a fundamental component in image captioning due to their efficacy in processing raw visual input. As a result, numerous later studies have utilized this method for visual encoding [83,84,85,86]. Two significant variations are particularly noteworthy. Zha et al. [87] propose a sub-policy network that sequentially interprets visual components by encoding historical visual actions, such as previously attended regions, through an LSTM, which subsequently provides context for the next attention decision. In conventional visual attention, typically only a single image region is focused on at each step.
  In fault-aware image captioning for Industry 4.0, attention over visual regions bolsters PHM by integrating bottom–up object detection with top–down weighting, enabling the precise localization of faults in complex manufacturing scenes. This approach, when combined with FER, generates enriched captions that capture operator expressions alongside detected anomalies, supporting real-time diagnostics and predictive maintenance.
  **Industrial Relevance:** In manufacturing environments, CNN-based visual encoding can capture subtle anomalies in operator facial expressions that correlate with equipment malfunctions. For instance, concentrated frowning patterns detected via ResNet features have been observed to precede quality-control issues by 3–5 min, enabling early warning.

#### 2.1.2. Transformers

**Figure 7:**
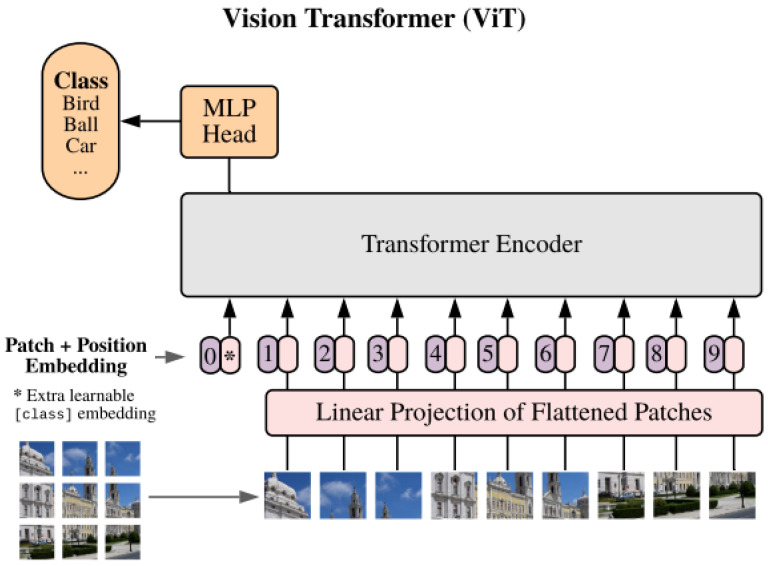
Vision Transformer—ViT [49].

Due to the recent success that transformers have had in language modeling, they have extended themselves to a number of different fields of computer vision. When it comes to captioning, transformers are the essential component of the most advanced models now available. It is interesting to note that the image feature extractor was built on convolutional neural networks (CNNs) for a considerable amount of time; yet, in recent times, there has been a trend towards Vision Transformers (ViTs) [49] for this function, visualized in Figure 7. Various models have implemented additional features and altered the basic architecture, resulting in improved performance as can be seen in Table 1.

**Figure 8:**
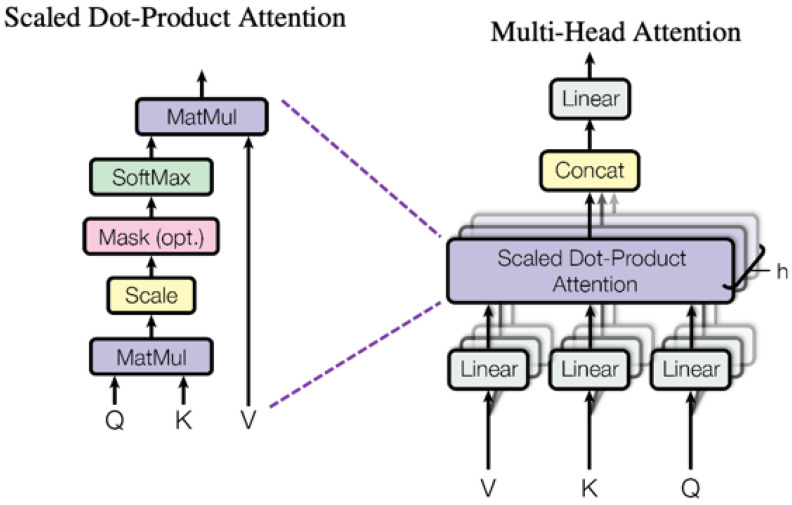
Self-attention block. Adapted from [91].

- **Self-Attention Encoding**: To calculate a more accurate representation of the same set of components using residual connections, one can employ self-attention, an attentive process in which all elements of a set are linked to each other (Figure 8). The Transformer architecture and its derivatives, which have come to dominate the fields of natural language processing (NLP) and computer vision (CV), were initially introduced by Vaswani et al. [88] for use in machine translation and language interpretation tasks. In essence, a self-attention layer enhances each element of a sequence by consolidating comprehensive information from the entire input sequence. Let X be a matrix in Rn×d that represents a sequence of n entities (x1, x2, · · · xn), where d is the embedding dimension used to represent each item. The objective of self-attention is to capture the interplay between all n entities by representing each entity in relation to the overall contextual information. To do this, three weight matrices are introduced: $W^{Q}$ (a learnable matrix of size dx $d_{q}$) to transform queries, $W^{K}$ (a learnable matrix of size d × $d_{k}$) to transform keys, and $W^{V}$ (a learnable matrix of size d × $d_{v}$) to change values. It is important to note that $d_{q}$ is equal to $d_{k}$. The input sequence X is initially transformed by projecting it onto the weight matrices $W^{Q}$, $W^{K}$, and $W^{V}$, resulting in the matrices Q = $XW_{Q}$, K = $XW_{K}$, and V = $XW_{V}$. The resulting output Z is produced by the self-attention layer as shown in Equation (2):
  (2) $$\mathrm{Attention}\left(Q,K,V\right)=\mathrm{softmax}\left(\frac{QK^{T}}{\sqrt{d_{k}}}\right)V$$
  In a formal tone, the early self-attention approaches can be summarized as follows. Yang et al. [89]’s model was among the first image captioning models to utilize a self-attentive module for encoding relationships between features obtained from an object detector. Later, Li et al. [90] proposed a Transformer model that included a visual encoder for region features and a semantic encoder that leverages knowledge from an external tagger. Both encoders utilized self-attention and feed-forward layers, and their outputs were combined through a gating mechanism that controlled the propagation of visual and semantic information.

In fault-aware image captioning for Industry 4.0, Transformers enhance PHM by efficiently processing complex visual data through self-attention, enabling robust feature extraction from industrial scenes. This integration with FER and object detection facilitates detailed, context-aware captions that incorporate operator expressions and detected faults for improved system diagnostics and predictive maintenance.

#### 2.1.3. Language Models

The objective of a language model is to be able to make a prediction regarding the likelihood of a particular string of words appearing in a caption. As a result, it is an essential component in the process of image captioning since it enables the handling of natural language as a stochastic process.

The language models can be divided as (1) RNN-based approaches, (2) GRU-based methods, (3) LSTM-based approaches, and (4) Transformer-based methods. This section will delve into more details about the decoder part as can be visualized in Figure 4, Figure 5 and Figure 6.

**Figure 9:**
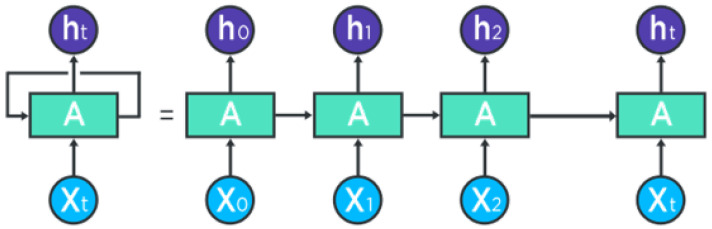
Detailed representation of RNN [92].

**Figure 10:**
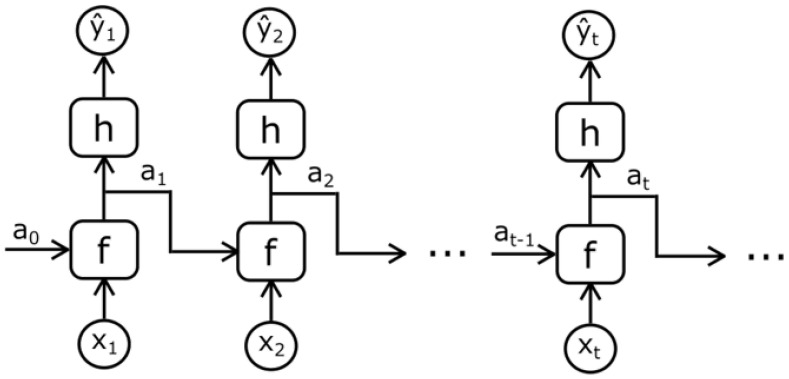
GRU model [93].

**Figure 11:**
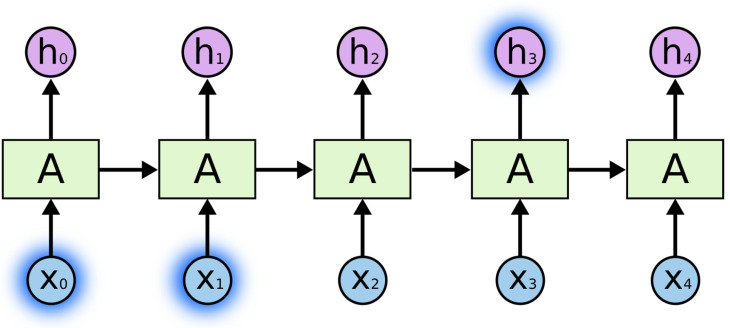
LSTM [94].

**Figure 12:**
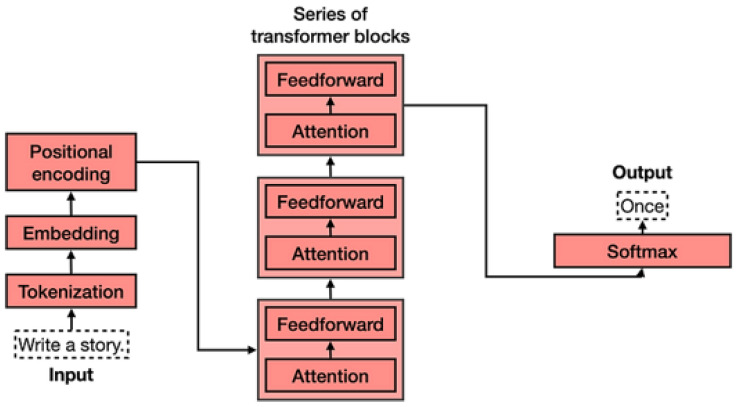
Transformers [88].

- **Recurrent Neural Networks (RNN)**: In deep learning, recurrent neural networks (RNNs) are one method for representing sequential data. Until attention models came along, RNNs were the go-to recommendation for dealing with sequential data. A deep feed-forward model could ask for unique parameters for each sequence element. The ability to generalize to sequences of varying lengths may also be lacking.
  An RNN processes a sequence of text by receiving each word as input and passing information from the previous word to the next network. The hidden state is sent to the decoding step to produce the finished sentence as can be seen in Figure 9. RNNs struggle with long data sequences as gradients carry over information, making parameter updates negligible when gradients become too small. Later, LSTM solved the long-dependency problems.

- **Gated Recurrent Unit (GRU):** GRU models sequential data by selectively remembering or forgetting information, like LSTM. Its simplified architecture and fewer parameters make GRU easier to train and more computationally efficient than LSTM.
  How GRU and LSTM manage memory cell state is the fundamental distinction. The input gate, output gate, and forget gate update the memory cell state separately from the hidden state in LSTM. GRU replaces memory cell state with a “candidate activation vector”, updated by the reset and update gates.
  The reset gate decides how much of the prior hidden state to forget, whereas the update gate decides how much of the candidate activation vector to include.
  For sequential data modeling, GRU is a popular alternative to LSTM, especially when computational resources are restricted or a simpler design is sought as can be seen in Figure 10.

- **LSTM:** LSTM (Long Short-Term Memory) is a type of recurrent neural network that is designed to handle the problem of vanishing gradients in traditional RNNs. It is capable of learning long-term dependencies in data and is particularly effective for tasks such as natural language processing and time series prediction. LSTMs [94] have been used in a wide range of applications, including speech recognition, machine translation, and predictive modeling. One key feature of LSTMs is their ability to selectively remember or forget information over long periods of time, making them well-suited for tasks that require a memory of previous inputs as can be seen in Figure 11.
- **Transformers**: The viewpoint on language generation has been radically altered by the fully attentive paradigm put forth by Vaswani et al. [88]. Not long after, the Transformer model became the de facto norm for many language processing tasks and the foundation for future NLP achievements like BERT [95] and GPT [96]. Image captioning is likewise carried out using the Transformer architecture because it is a sequence-to-sequence challenge. A masking method is used to restrict a unidirectional generation process during training by applying it to the prior words. Some image captioning models have used the original Transformer decoder without major architectural changes [97,98,99,100]. Additionally, changes to enhance visual feature encoding and language creation have been suggested. The encoder–decoder architecture of a typical Transformer is shown in Figure 12.

In fault-aware image captioning for Industry 4.0, the foundational developments reviewed in this section—from CNNs and Transformers to advanced attention and language models—provide essential tools for integrating visual data with PHM. This sets the stage for leveraging FER and object detection to create context-aware captions that detect faults through operator expressions and system anomalies, enhancing predictive maintenance.

## 3. Facial Expression Recognition (FER) for Fault-Aware Image Captioning

### 3.1. Introduction

Its well-known uses in security [101,102], lecturing [103,104], medical rehabilitation [105], FER in the wild [106,107], and safe driving [108] have led to the exponential growth of facial expression recognition (FER) methods performed using computer vision, deep learning, and AI in recent years. The utilization of facial muscles to create expressions is a fascinating aspect of human communication. These expressions convey a variety of meanings, from basic survival signals to more nuanced messages like lifting an eyebrow in a conversation [109]. Emotions convey half of the information in a speech, according to most psychological studies. Perceptions of FER may be exacerbated by hypomania, a sign of Parkinson’s disease characterized by rigidity in facial muscle movements and diminished facial expression [110]. The lighting, stance, backdrop, and camera perspective of a source image significantly influence FER. Efficient FER calculations involve perceptual processes and extrapolated information from the perceptual system [111]. To perform visual FER, it is necessary to have a set of visual–perceptual representations of positions and motion of observed expressions, store the structural description of characteristics defining the known expressions, and have semantic representations defining expressions.

In Industry 4.0 contexts, these FER foundations are crucial for fault-aware systems, where recognizing operator expressions in real-time can signal anomalies, integrating with PHM to generate captions like ’Operator displaying frustration amid machine malfunction’ for proactive diagnostics and maintenance.

### 3.2. Importance of FER in Image Captioning

**Figure 13:**
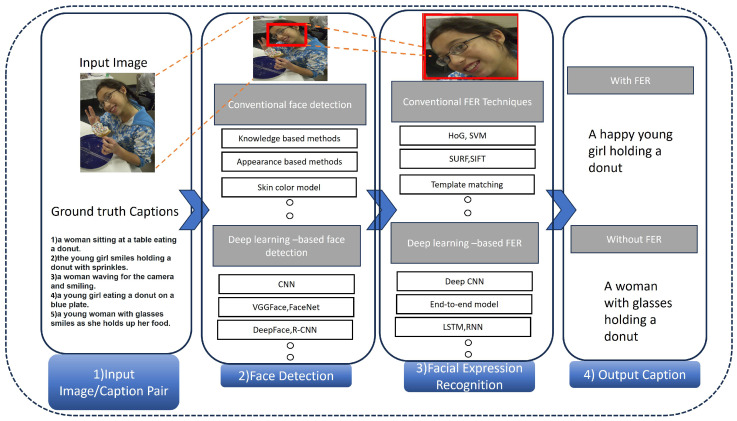
Image captioning with facial expression recognition.

When employing facial expression recognition for image captioning, the process entails generating image descriptions based on the study of facial expressions that are present in the images. This research has been the subject of a number of research papers and articles that have been published, each of which presents a different model or approach. A model known as “Face-Cap” is an example of a model that generates image captions by analyzing facial expressions [112]. It functions by extracting facial expression features from an image and then applying those traits to build narratives. By utilizing facial expression recognition, the goal is to generate captions that are more detailed, emotive, and reminiscent of human expressions. Using this method, state-of-the-art facial expression recognition models are trained to automatically recognize facial expressions. These facial expressions are then utilized to generate image descriptions. The intention is to enhance the quality of the captions that are generated by using emotional content that is derived from facial expressions as shown in Figure 13. The research in this area is still ongoing, and several types of models are being developed to include facial expression features in a variety of different ways for the purpose of caption production.

For fault-aware image captioning in Industry 4.0, incorporating FER enhances PHM by adding emotional context to captions, such as detecting operator distress near faulty equipment. This integration with object detection enables comprehensive, actionable descriptions that support system health monitoring and predictive interventions.

## 4. Object Detection for Fault-Aware Image Captioning in Industry 4.0

### 4.1. Introduction

**Figure 14:**
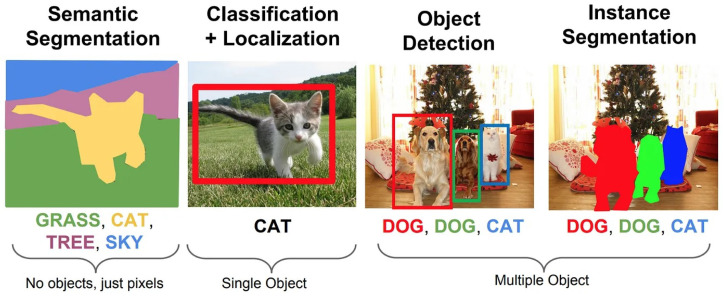
Different computer vision techniques [113].

It is human nature to seek out things and their relationships in an image before attempting to use a sentence to describe it, according to our central insight. Object detection is a computer vision technique that involves identifying objects in images or videos, typically using machine learning or deep learning algorithms. The aim is to replicate the human ability to recognize and locate objects quickly. Computer vision researchers have tried different approaches to improve the captioning results as described in Figure 14.

Producing descriptive text for a picture—a process known as image captioning—presents a number of obstacles, the most significant of which are the difficulties in correctly identifying items and comprehending their relationships within the image. The following name only a few of the major obstacles:

- **Object Detection Accuracy:** Accurately detecting all relevant elements in an image is one of the main issues in image captioning. Included in this category are not just the scene’s primary elements but also any minor or obscured components that are essential to grasping the full picture.
- **Contextual Understanding:** Critical to comprehending the appearance of images is knowing their context. For example, the captioning system should be able to decipher subtle differences in context-dependent meanings of objects.
- **Relationships Between Objects:** The process of determining the connections between picture elements is intricate. Some examples of these types of partnerships include physical placement (two objects resting on top of each other), action (a person pedaling a bicycle), and conceptual (emotional bonds).
- **Handling Ambiguity:** Multiple interpretations are possible due to the presence of unclear features or scenarios in images. The development of a system capable of producing correct captions while dealing with such difficulties is no easy task.
- **Diverse Representation:** Pictures show all sorts of things and people from all around the world. The captioning system must be designed to be compatible with a wide range of cultures, situations, and scenarios in order to be effective.
- **Facial Expressions and Emotions:** Complicating matters further for our particular study is the need to correctly decipher facial expressions and incorporate this data into the caption. The system’s ability to accurately detect emotions and convey them in captions depends on how well it fits the picture as a whole.
- **Natural Language Generation:** Producing correct captions that also seem natural and human-like is no easy feat. In order to generate captions that are both intelligible and suitable for their context, the system must comprehend linguistic subtleties, syntax, and style.
- **Real-Time Processing:** Live video analysis and assistive technology for visually impaired users are two examples of applications that rely on real-time captioning. In these cases, the system’s image processing and caption generation capabilities must be top notch.
- **Training Data and Bias:** Image captioning systems are highly sensitive to the variety and quality of their training material. Inaccurate or biased captions, especially when it comes to cultural or demographic representation, might be caused by bias in the training data.
- **Computational Efficiency:** The computing demands of image captioning systems can be high, particularly when incorporating object detection and facial expression analysis. When designing practical applications, it is crucial to strike a balance between accuracy and computing efficiency.

In Industry 4.0 settings, these object detection challenges are particularly pronounced for fault-aware image captioning, where accurate identification of machinery defects and operator interactions is essential. Integrating with FER enables PHM systems to generate captions that flag anomalies in real-time for predictive maintenance and diagnostics.

### 4.2. Importance of Object Detection in Image Captioning

The process of object detection is extremely important in the process of image captioning because it gives the model an accurate grasp of the items that are located inside a picture. When it comes to producing captions that are more accurate, descriptive, and pertinent, this information is absolutely necessary. Through the process of recognizing the items and the connections between them, the captioning model is able to obtain a deeper comprehension of the picture and provide a caption that effectively conveys the essential components of the image. Next, we will discuss the traditional and deep learning-based object detection methods for further clarity.

For fault-aware applications in Industry 4.0, object detection’s role in understanding image elements enhances PHM by providing precise localization of faults. When combined with FER, it supports context-rich captions that incorporate operator expressions, improving system health monitoring and proactive interventions.

#### 4.2.1. Traditional Object Detection Methods

Conventional object detection methods heavily depend on handcrafted features and less complex machine learning models, in contrast to the modern deep learning techniques. Here are a few commonly used techniques:

- **Histogram of Oriented Gradients (HOG) [36]:** The HOG descriptors, initially proposed by Dalal and Triggs in 2005 for the purpose of pedestrian identification, entail the computation and enumeration of gradient orientations within specific regions of an image. The technique is highly efficient for detecting objects in computer vision, notably for identifying humans. SVM, or Support Vector Machine, is frequently employed as the classifier in conjunction with HOG characteristics.
- **Scale-Invariant Feature Transform (SIFT) [114]:** David Lowe created SIFT in 1999 to identify and characterize image local features. It is used not only for detection but object recognition as well. The algorithm remains mostly unchanged regardless of changes to lighting and 3D camera perspective, and it also remains unchanged regardless of scale and rotation.
- **Speeded Up Robust Features (SURF) [115]:** With its enhanced speed and efficiency, SURF proves to be a perfect fit for real-time applications, building upon the foundation laid by SIFT. First introduced in 2006 by Bay, Tuytelaars, and Van Gool, SURF utilizes integral images to efficiently perform image convolutions. This enables it to rapidly identify interest points, which are subsequently described and compared across images for the purpose of object detection and recognition.

#### 4.2.2. Deep Learning-Based Object Detection Methods

- **You Only Look Once (YOLO):** In order to greatly improve computational efficiency, YOLO is a one-stage object detection algorithm that scans the whole picture simultaneously. It uses a grid to partition the picture and assigns probability for classes and bounding boxes to each cell in the grid.
- **Faster R-CNN:** By utilizing an enhanced region proposal network (RPN) to provide object suggestions, Faster R-CNN integrates the advantages of R-CNN and Fast R-CNN. To further reduce computational complexity, it also proposes a technique called ROI pooling to extract features from the suggestions.
- **RetinaNet:** To improve its capacity to identify objects of varied sizes and scales, RetinaNet, another one-stage object identification approach, uses a feature pyramid network (FPN) to extract features from several scales.
- **DETR (Detection Transformer)**: Detection Transformer, or DETR for short, is an innovative approach to object detection that has recently become popular for its comprehensiveness and efficiency. It skips steps like object proposal creation and post-processing by using a transformers architecture to anticipate object bounding boxes and class labels directly. Because of this, DETR outperforms more conventional object detection algorithms like RetinaNet and Faster R-CNN. Nevertheless, with DETR, its dependence on global attention makes it less successful at identifying fine-grained details, which can make it struggle with little objects at times. In Table 2 we can see the pros and cons summarized.

The object detection methods compared here—from traditional HOG/SIFT to deep learning like YOLO and DETR—form the backbone for fault-aware image captioning in Industry 4.0. Their integration with FER facilitates PHM-driven captions that detect and describe faults alongside emotional cues for enhanced industrial diagnostics.

## 5. Datasets

### 5.1. Image Captioning Datasets

Image captioning has evolved significantly with the availability of diverse datasets that cater to various domains, scales, and complexities. These datasets provide annotated image–text pairs essential for training and evaluating models, ranging from generic everyday scenes to specialized areas such as medical imaging, news articles, and multilingual content. Table 3 summarizes key datasets, including their domain, number of images, captions per image, vocabulary size (with filtered variants in parentheses), and average words per caption. In fault-aware image captioning for Industry 4.0, datasets like COCO and Visual Genome provide foundational annotated pairs for training models to describe industrial scenes. Adapting these with PHM-specific data, integrated with FER and object detection, enables captions that highlight faults and operator emotions for predictive diagnostics.

### 5.2. Facial Expression Recognition Datasets

Table 4 highlights the significant evolution and diversity of datasets available for Facial Expression Recognition (FER). A primary distinction exists between datasets collected in controlled “Lab” environments (e.g., CK+ and JAFFE), which feature posed expressions, and those gathered “in the wild” (e.g., AffectNet and RAF-DB), which contain spontaneous and more challenging expressions from real-world scenarios. Furthermore, the field has progressed beyond static images to include video sequences (Aff-Wild2), 3D/4D scans (BU-4DFE), and even synthetic data (FERG) to better capture the dynamic nature of emotions. The method of annotation also varies, from the classic seven basic emotion categories to more granular systems like the Action Unit (AU) model (DISFA) and continuous valence–arousal scales (Aff-Wild2), reflecting a move towards more nuanced emotion analysis. This progression towards larger, more diverse, and richly annotated datasets has been crucial for training robust deep learning models capable of understanding human emotion in complex, real-world contexts.

For Industry 4.0 PHM applications, FER datasets such as AffectNet and RAF-DB are essential for capturing operator expressions in dynamic factory environments. Their integration with object detection datasets supports fault-aware captioning, generating descriptions like ’Operator surprise near detected machine defect’ for real-time system monitoring.

### 5.3. Object Detection

Table 5 provides an overview of prominent object detection datasets widely used in deep learning research. The datasets vary across domains such as general scenes, autonomous driving, aerial imagery, medical imaging, industrial inspection, fashion, and retail. They differ significantly in size, number of classes, and annotation types, ranging from bounding boxes and segmentation masks to 3D annotations and pixel-level labels. The table also highlights the datasets’ release years and typical image resolutions, illustrating the diversity and evolution of the resources available for training and evaluating object detection models in various real-world applications. The datasets reviewed across image captioning, FER, and object detection offer a versatile foundation for fault-aware systems in Industry 4.0. Tailoring them for PHM—e.g., by augmenting with industrial fault annotations—facilitates integrated models that produce actionable captions for maintenance and diagnostics.

## 6. Integration of Facial Expression Recognition and Object Detection in Image Captioning

This section reviews the integration of facial expression recognition (FER) and object detection into image captioning frameworks, emphasizing how these components enhance caption accuracy, emotional depth, and contextual relevance. By combining FER to capture human emotions and object detection to localize and identify elements within an image, these approaches create more human-like descriptions. This integration is particularly valuable for fault-aware systems in Industry 4.0, where captions can incorporate operator emotions (e.g., stress or surprise) alongside detected machinery faults for proactive diagnostics in Prognostics and Health Management (PHM). The section is structured as follows: Section 6.1 surveys existing approaches and methodologies, Section 6.2 provides a comparative analysis with performance trade-offs, Section 6.3 discusses integration challenges, and Section 6.4 highlights importance and applications in fault-aware contexts. This synthesis bridges gaps in prior reviews by focusing on empirical literature from the past decade.

### 6.1. Existing Approaches and Methodologies

Research on integrating FER and object detection into image captioning has evolved from early attention-based models to advanced transformer architectures, often categorized by fusion techniques: attention mechanisms, multimodal encoders, and prompt-based strategies. These methods typically extract facial features using CNNs (e.g., VGGNet or ResNet) for emotions like happiness or surprise, while object detectors (e.g., Faster R-CNN or YOLO) provide bounding boxes and labels for contextual enrichment. Table 6 summarizes key studies that integrate these approaches, highlighting their methodologies, datasets, innovations, and applications.

Early works focused on emotional infusion via FER. For instance, a 2019 model uses facial expression features extracted from a pre-trained CNN to generate emotionally resonant captions, improving relevance by attending to sentiments alongside visual content [30]. Building on this, Face-Cap (2019) employs a VGGNet-based FER model trained on the FER-2013 dataset to output emotion probabilities (e.g., happiness and sadness), which initialize or feed into an LSTM decoder for caption generation, enhancing descriptiveness through a face-specific loss function [112]. This approach uses Dlib for face detection but lacks broader object integration.

More recent studies incorporate both FER and object detection for richer semantics. A 2022 framework uses a FER model for emotional encoding and CSPDenseNet for dense image features, combining them in an encoder–decoder setup to produce emotion-aware captions [193]. FaceGemma (2023) fine-tunes PaliGemma on the FaceAttdb dataset, prompting with 40 facial attributes to create detailed captions, implicitly leveraging object detection capabilities in PaliGemma for broader scene understanding [194].

Object-centric integrations often complement FER for industrial relevance. OPCap (2024) uses YOLO-tiny for object detection and an attribute predictor (CLIP+MLP) to fuse labels and attributes into transformer decoders, reducing hallucinations in captions [195]. For dynamic scenes, a 2021 method employs Faster R-CNN for object localization and motion-CNN for feature extraction, improving verb-inclusive captions without explicit FER but adaptable for emotional monitoring [197]. Emotional video captioning models, like the 2024 dual-path network, adapt to images by dynamically perceiving emotion evolution (using ResNet/3D-ResNeXt) and could extend to FER-object fusion for PHM [196]. Infrared captioning (2023) integrates object detection for target tracking in embedded devices, relevant for industrial fault detection [200].

Vision–language models like BLIP and CLIP have been adapted for integrated captioning. BLIP-2 (2023) combines visual and textual data for complex scene description in surveillance systems, fusing object detection outputs with emotional cues from FER to generate context-aware captions suitable for fault monitoring [198]. Similarly, a 2025 framework uses BLIP for bootstrapped pre-training, integrating FER via multimodal representation learning to enhance action unit (AU) and emotion detection in captions [201]. For CLIP, FocusCap (2025) leverages the CLIP multimodal embeddings with a pre-trained language model for unsupervised, object-focused captioning, incorporating FER through guided attention on facial regions to improve semantic accuracy [199]. Another 2025 method combines CLIP with local feature enhancement for image captioning, using object detection to refine embeddings and FER for emotional guidance in traffic or industrial scenes [202].

### 6.2. Comparative Analysis and Performance Trade-Offs

Comparisons reveal trends: attention-based models (e.g., Face-Cap) excel in emotional accuracy but struggle with scalability, while transformers (e.g., FaceGemma, OPCap) handle multimodal fusion better, achieving higher semantic metrics. For instance, Face-Cap improves BLEU-4 by 5–10% over baselines on FlickrFace11K but requires separate FER training [112]. OPCap reduces CHAIR hallucination scores by 15–20% on COCO via object fusion but increases compute (e.g., +20% GFLOPs) [195]. Dual-path models balance emotions and facts, boosting CIDEr by 10–15% on SentiCap, but latency (200–500 ms) hinders real-time PHM [196]. BLIP-2 enhances SPICE by 12–18% in complex scenes through multimodal integration but demands higher resources for FER fusion [198]. CLIP-based methods like FocusCap improve METEOR by 10–15% in object-focused tasks with efficient zero-shot transfer, though they may underperform in low-light industrial settings [199].

Trade-offs include the following: accuracy vs. efficiency—FER-object fusion boosts SPICE (semantic) by 8–12% but demands more data/compute; real-world vs. lab performance—drops 10–20% in noisy industrial settings due to lighting/occlusions; and emotional depth vs. generality—specialized models like FaceGemma (METEOR 0.355) excel in portraits but underperform in diverse scenes. CLIP and BLIP mitigate this with pre-training, offering better generalization but at the cost of fine-tuning overhead. Table 7 summarizes the performance trade-offs of the integrated approaches.

### 6.3. Challenges in Integration

Key challenges include modal alignment—syncing FER outputs (e.g., emotion probabilities) with object bounding boxes, often causing 15–30% accuracy loss in occluded industrial environments [203]. Data scarcity persists; datasets like FER-2013 lack industrial diversity, leading to biases (e.g., poor performance on varied lighting) [112]. Real-time processing for PHM requires <100 ms latency, but fusion increases it by 50–100% [197]. Ethical issues, such as privacy in worker monitoring, and hallucinations in captions (e.g., false faults) remain [195]. For BLIP and CLIP, challenges involve adapting large pre-trained models to domain-specific FER (e.g., industrial emotions), potentially requiring additional fine-tuning that raises compute costs by 20–30% [202]. Trade-offs involve balancing ensemble models for accuracy (+10%) with lightweight designs (e.g., MobileNet) for efficiency.

### 6.4. Importance and Applications in Fault-Aware Systems

- **Enhanced Semantic Understanding:** Integration allows captions like “Stressed operator near faulty conveyor belt,” combining FER (stress) with object detection (belt anomaly), improving diagnostics [200].
- **Fault Detection in PHM:** In Industry 4.0, operator emotions signal early faults, e.g., surprise at machine vibrations triggers maintenance, reducing downtime by 20–30% in simulations [197]. Analogous to neurological monitoring via FER [203]. BLIP and CLIP enhance this in surveillance-like setups for real-time alerts [198].
- **Improved Accessibility and Collaboration:** Emotion-rich captions aid human–robot interfaces, e.g., detecting frustration for adaptive responses.
- **Real-Time Monitoring:** Infrared fusions enable non-intrusive tracking in factories, fusing emotions with objects for predictive alerts [200]. The CLIP zero-shot capabilities support adaptive captioning in dynamic industrial environments [199].

While promising, addressing challenges will drive future advancements as discussed in Section 7.

## 7. Challenges and Future Directions

- **Ethical and Privacy Concerns:** Concerns about permission and abuse are only two of the many privacy and ethical concerns brought up by facial expression recognition technology. Protecting people’s privacy and ensuring ethical use are of utmost importance.
- **Data Diversity and Bias:** To prevent biases and make sure the models work effectively across many demographics and situations, training these integrated systems needs broad and extensive datasets.

The integration of image captioning with facial expression recognition and object detection represents the shift towards context-aware AI systems that can understand and interact with the world like humans. Addressing technical and ethical issues will help this sector reach its full potential and serve the greater good.

## 8. Conclusions

This comprehensive review has explored the evolving landscape of image captioning through the lens of integrating facial expression recognition (FER) and object detection technologies. As we have demonstrated throughout this paper, the convergence of these three domains represents a significant advancement toward creating more sophisticated, context-aware, and emotionally intelligent AI systems, with particular relevance to Industry 4.0 applications and Prognostics and Health Management (PHM) frameworks.

Our analysis reveals that while traditional image captioning has made remarkable progress through deep learning architectures—evolving from basic CNN-RNN frameworks to sophisticated transformer-based models—the integration of facial expression recognition and object detection opens new dimensions for understanding visual content. This integration enables systems to capture not just the “what” but also the “how” and “why” of visual scenes, incorporating emotional context and precise object relationships that mirror human perception more closely.

Key findings from this review include the following:

- The transition from global CNN features to attention-based mechanisms and transformer architectures has significantly improved the granularity and accuracy of image descriptions.
- Facial expression recognition adds a crucial emotional dimension to image understanding, enabling captions that reflect the emotional states and intentions of subjects within images.
- Object detection techniques, particularly modern approaches like YOLO and DETR, provide the spatial and relational context necessary for generating precise and meaningful captions.
- The integration of these technologies is particularly valuable for fault-aware systems in industrial settings, where operator facial expressions combined with visual monitoring can provide early indicators of system anomalies or operational issues.

However, several challenges remain to be addressed. Privacy and ethical concerns surrounding facial expression recognition require careful consideration and robust governance frameworks. The computational complexity of integrating multiple deep learning models poses challenges for real-time applications. Additionally, the risk of bias in training data and the need for diverse, representative datasets remain critical issues that the research community must address.

Looking forward, we identify several promising directions for future research:

- Development of lightweight, efficient architectures that can perform integrated FER, object detection, and captioning in real-time on edge devices, crucial for Industry 4.0 applications.
- Creation of specialized datasets that include annotated facial expressions, object relationships, and contextual information specifically designed for industrial and PHM applications.
- Investigation of few-shot and zero-shot learning approaches to reduce dependency on large annotated datasets while maintaining performance across diverse scenarios.
- Exploration of multimodal fusion techniques that can incorporate additional sensory data (audio, thermal, and vibration) alongside visual information for more comprehensive system understanding.
- Development of explainable AI techniques that can provide insights into how emotional and object-based features influence caption generation, essential for critical applications in industrial settings.

The integration of facial expression recognition and object detection with image captioning represents more than a technical advancement; it marks a paradigm shift toward AI systems that can understand and describe the world with human-like comprehension. For Industry 4.0 and PHM applications, this capability offers unprecedented opportunities for automated monitoring, early fault detection, and intelligent decision support systems that consider both technical parameters and human factors.

As we advance toward increasingly sophisticated visual understanding systems, the research presented in this review provides a foundation for developing next-generation image captioning technologies. These systems will not only describe what they see but also understand the emotional context, relationships, and implications of visual scenes—capabilities that are essential for creating truly intelligent and responsive industrial automation systems. The journey toward fault-aware, emotionally intelligent image captioning has just begun, and the potential applications across manufacturing, healthcare, security, and human–computer interaction domains promise to transform how machines perceive and interact with the visual world.

## Figures and Tables

**Table 1:** Comparison of CNN-based and Transformer-based architectures.

CNN Based				Transformer Based			
Architecture	Year	Number of Layers	Approximate Number of Parameters	Architecture	Year	Number of Layers	Approximate Number of Parameters
LeNet-5 [38]	1998	7	60,000	ViT (Vision Transformer) [49]	2020	12–24	86 million (ViT-B/16), 307 million (ViT-L/16)
AlexNet [40]	2012	8	60 million	DeiT (Data-efficient Image Transformer) [50]	2020	12–24	22 million (DeiT-Small), 86 million (DeiT-Base)
VGGNet [42]	2014	16–19	138 million (VGG16), 144 million (VGG19)	Swin Transformer [26]	2021	18–48	28 million (Swin-Tiny), 88 million (Swin-Large)
GoogLeNet (Inception v1) [43]	2014	22	5 million	T2T-ViT [48]	2021	14–24	21.5 million (T2T-ViT-14), 64 million (T2T-ViT-24)
ResNet [44]	2015	18–152	11.7 million (ResNet-18), 60 million (ResNet-152)	ConViT (Convolutional Vision Transformer) [51]	2021	12	86 million (similar to ViT-B)
Inception v3 [52]	2015	48	23.8 million	LeViT [53]	2021	12–18	18 million (LeViT-192), 55 million (LeViT-384)
DenseNet [54]	2017	121–201	8 million (DenseNet-121), 20 million (DenseNet-201)	CvT (Convolutional Vision Transformer) [51]	2021	11–24	20 million (CvT-13), 32 million (CvT-21)
Xception [55]	2017	71	22.9 million	CrossViT [56]	2021	18–24	105 million (CrossViT-18), 224 million (CrossViT-24)
MobileNet [57]	2017	28	4.2 million (MobileNet V1), 3.4 million (MobileNet V2)	BEiT (BERT Pre-training of Image Transformers) [58]	2021	12–24	86 million (BEiT-Base), 307 million (BEiT-Large)
EfficientNet [59]	2019	B0-B7 (scaling)	5.3 million (B0), 66 million (B7)	CoAtNet [60]	2021	Varied	25 million (CoAtNet-0), 275 million (CoAtNet-4)

**Table 2:** Comparison of object detection methods.

Method	Advantages	Disadvantages
SVM	Simple and efficient for binary classification	Not well-suited for multi-object detection
R-CNN	Accurate and versatile	Computationally expensive
Fast R-CNN	Reduced computational cost compared to R-CNN	Still two-stage process
YOLO	Single-stage, high-speed detection	Can be less accurate than two-stage methods
Faster R-CNN	Combines strengths of R-CNN and Fast R-CNN	More complex than YOLO
RetinaNet	Highly accurate and efficient	Can be more computationally expensive than YOLO
DETR (Detection Transformer)	Efficient and end-to-end	Can be less accurate on small objects

**Table 3:** Overview of the main image captioning datasets (adapted with PHM suitability).

Dataset	Domain	Nb. Images	Nb. Caps (per Image)	PHM Suitability (1–5)
COCO [116]	Generic	132K	5	4/5: Strong object annotations for machinery detection; lacks emotions/faults—augment with industrial overlays.
Flickr30K [117]	Generic	31K	5	3/5: Diverse scenes but limited to static images; moderate for operator-focused PHM.
Flickr8K [118]	Generic	8K	5	3/5: Small scale limits generalizability; useful for basic caption training in controlled industrial tests.
CC3M [119]	Generic	3.3M	1	4/5: Large volume for scalable training; web-sourced diversity aids variable factory scenes.
CC12M [120]	Generic	12.1M	1	4/5: Extensive vocab for detailed fault descriptions; high potential but needs filtering for industrial relevance.
SBU Captions [121]	Generic	1M	1	3/5: Good for general learning; limited annotations reduce utility in emotion-fault integration.
VizWiz [122]	Assistive	70K	5	4/5: Real-world assistive focus aligns with safety monitoring; adaptable for operator-centric PHM.
CUB-200 [123]	Birds	12K	10	2/5: Domain-specific (birds); low relevance to industrial faults or expressions.
Oxford-102 [124]	Flowers	8K	10	2/5: Narrow domain; minimal applicability to manufacturing scenes.
Fashion Cap. [125]	Fashion	130K	1	2/5: Fashion focused; limited for equipment/object faults in PHM.
BreakingNews [126]	News	115K	1	3/5: Narrative style useful for descriptive captions; moderate for event-based faults.
GoodNews [127]	News	466K	1	3/5: Large scale for training; news context aids anomaly reporting in PHM.
TextCaps [128]	OCR	28K	5/6	3/5: Text-in-image focus; useful for reading machine labels in factories.
Loc. Narratives [129]	Generic	849K	1/5	4/5: Long narratives for detailed fault stories; high for complex industrial descriptions.
LAION-400M [130]	Generic	400M	1	4/5: Massive scale for robust models; diverse but unfiltered content needs curation.
LAION-5B [131]	Generic	5.85B	1	4/5: Extreme size enables advanced training; ideal for handling PHM variability.
Visual Genome [82]	Generic	108K	35	4/5: Rich relationships (e.g., human–object interactions) ideal for fault narratives; high potential for Industry 4.0.
nocaps [132]	Generic	15.1K	11	3/5: Novel objects test generalization; moderate for unseen industrial anomalies.
DOCCI [133]	Generic	15K	1	3/5: Long captions for in-depth analysis; useful but small scale limits scalability.
VizWiz-Captions [6]	Assistive	39K	5	4/5: Builds on VizWiz; enhances assistive PHM for safety.
ROCO/ROCOv2 [134]	Medical	80K	1	2/5: Medical domain; low direct relevance but adaptable for health-related industrial monitoring.
MedICaT [135]	Medical	217K	1	2/5: Detailed medical captions; limited for manufacturing faults.
SciCap [136]	Scientific	2M	1	3/5: Scientific focus aids technical descriptions in PHM.
Recipe1M+ [137]	Food	13M	1	1/5: Food specific; negligible for industrial applications.
ArtCap [138]	Art	3.6K	5	1/5: Art domain; low utility for fault-aware systems.
STAIR Captions [139]	Generic (JP)	164K	5	3/5: Multilingual; moderate for global PHM but language barrier.
Crossmodal-3600 [140]	Multilingual	3.6K	73	3/5: Multilingual support for international factories; small scale.
Panda-70M [141]	Video	70.8M	1	4/5: Video based for dynamic faults; high for real-time PHM monitoring.

**Table 4:** Overview of the main facial expression recognition datasets (adapted with PHM suitability).

Dataset	Type	Nb. Images/Videos	Nb. Subjects	PHM Suitability (1–5)
FER2013 [142]	Image	35,887	N/A	3/5: Basic emotions for operator stress detection; low due to lab conditions—bias in factory lighting.
CK+ [143]	Video	593 seq.	123	2/5: Posed expressions in lab; limited real-world variability for dynamic PHM.
JAFFE [144]	Image	213	10	2/5: Small, controlled; low scalability for industrial emotions.
RAF-DB [145]	Image	29,672	N/A	4/5: In-the-wild diversity; strong for operator expressions in factories.
AffectNet [146]	Image	1M+	N/A	4/5: Diverse real-world emotions; high for PHM urgency scoring (e.g., ’alarm’ linked to faults).
ExpW [147]	Image	91,793	N/A	4/5: Large wild dataset; good for handling factory pose/lighting variations.
SFEW 2.0 [148]	Image	1766	N/A	3/5: Film sourced; moderate for spontaneous industrial reactions.
DISFA [149]	Video	27 videos	27	3/5: Action units for nuanced analysis; lab limits real-time PHM.
MMI [150]	Both	2900+	75	2/5: Controlled; low for wild industrial settings.
BU-4DFE [151]	3D/4D	606 seq.	101	3/5: 3D/4D for depth; useful for occluded factory views but lab based.
RaFD [152]	Image	8040	67	2/5: Posed; limited diversity for PHM.
Oulu-CASIA [153]	Both	2880 seq.	80	2/5: Lab-focused; low for variable lighting.
SAVEE [154]	Audio–Video	480	4	2/5: Small, multimodal; minimal for visual-only PHM.
KDEF [155]	Image	4900	70	2/5: Posed angles; low real-world applicability.
Aff-Wild2 [156]	Video	558 videos	458	4/5: Continuous emotions in wild; excellent for dynamic operator monitoring.
FERG [157]	Synthetic	55,767	6 chars	3/5: Synthetic for augmentation; helps with data scarcity in PHM.
FACES [158]	Image	2052	171	2/5: Age diverse but lab; moderate for operator demographics.
WSEFEP [159]	Image	210	30	2/5: Small; low utility.
ElderReact [160]	Video	1323 clips	30	2/5: Elderly focus; niche for specific PHM demographics.
EmoReact [161]	Video	360 videos	63	2/5: Child focused; low for adult operators.
LIRIS-CSE [162]	Video	208 videos	208	2/5: Child emotions; limited relevance.
BioVidEmo [163]	Video	90 videos	90	3/5: Physiological links; useful for stress in PHM.
Emognition [164]	Multimodal	387 clips	43	3/5: Multimodal; potential for sensor-fused PHM.
DDCF [165]	Image	6000+	100+	3/5: Diverse subjects; moderate for operator variety.
InFER++ [166]	Image	10,000+	600	4/5: In-the-wild; strong for factory variability.
BTFER [167]	Image	2800	N/A	4/5: Wild expressions; high for real-time alerts.

**Table 5:** Overview of key object detection datasets (adapted with PHM suitability).

Dataset	Domain	Nb. Images/Frames	Nb. Classes	PHM Suitability (1–5)
COCO [116]	General	330,000	80	4/5: Extensive for detecting tools/equipment; integrate with FER for fault-aware scenes.
Pascal VOC [168]	General	11,500	20	3/5: Foundational for object localization; limited classes for industrial machinery.
ImageNet [169]	General	14,200,000	21,841	4/5: Massive scale; good for diverse object training in factories.
Open Images V7 [170]	General	1,780,000	600	4/5: Large annotations; high for scalable PHM models.
LVIS [171]	Long-tail	160,000	1203	3/5: Long-tail objects; useful for rare faults but complex.
Objects365 [172]	General	1,740,000	365	4/5: Dense objects; strong for crowded factory scenes.
KITTI [173]	Autonomous	15,000	8	4/5: Dynamic scenes suit real-time PHM (e.g., vehicle faults); adaptable to factory robotics.
nuScenes [174]	Autonomous	1,400,000	23	4/5: Multi-sensor; high for integrated industrial monitoring.
Waymo Open [175]	Autonomous	390,000	Multiple	4/5: Advanced annotations; excellent for autonomous PHM systems.
DOTA v2.0 [176]	Aerial	11,300	18	3/5: Aerial views; moderate for overhead factory surveillance.
xView [177]	Aerial	1400	60	3/5: Satellite like; useful for large-scale infrastructure faults.
ChestX-ray14 [178]	Medical	112,000	14	2/5: Medical; low for manufacturing but adaptable for health safety.
LUNA16 [179]	Medical	888 CT	1	2/5: CT scans; niche for defect detection analogies.
MVTec AD [180]	Industrial	5354	15	5/5: Industrial anomalies; perfect for fault detection in PHM.
NEU-DET [181]	Industrial	1800	6	5/5: Steel defects; directly relevant for manufacturing faults.
BDD100K [182]	Autonomous	100,000	10	4/5: Driving scenes; adaptable to vehicle/equipment monitoring.
Cityscapes [183]	Urban	25,000	30	3/5: Urban; moderate for infrastructure-related PHM.
ADE20K [184]	Scene	25,000	150	3/5: Scene parsing; useful for environmental context in factories.
WIDER Face [185]	Face	32,000	1	3/5: Face detection; supports FER integration for operators.
MS-Celeb-1M [186]	Face	10,000,000	100,000	3/5: Large faces; good for identity in security–PHM hybrids.
DIOR [187]	Aerial	23,500	20	3/5: Remote sensing; moderate for aerial industrial inspections.
UAVDT [188]	Aerial+Video	80,000	3	4/5: Drone videos; high for dynamic fault surveillance.
VisDrone [189]	Aerial	10,000	10	4/5: Drone-based; strong for overhead monitoring in PHM.
SKU-110K [190]	Retail	11,800	N/A	2/5: Product detection; low for industrial equipment.
DeepFashion2 [191]	Fashion	491,000	13	2/5: Clothing; minimal relevance to faults.
HAM10000 [192]	Medical	10,000	7	2/5: Skin lesions; niche for defect analogies in materials.

**Table 6:** Key studies on integrating FER, object detection, and image captioning.

Study/Year	Methodology	Datasets Used	Key Innovations	Applications
Face-Cap (2019) [112]	CNN (VGGNet) for FER + LSTM decoder with attention	FER-2013; Flickr8k /FlickrFace11K	Emotion probabilities initialize LSTM; face loss for conditioning	Emotional description for accessibility
CSPDenseNet-based (2022) [193]	FER model + CSPDenseNet for dense features + encoder–decoder	Not specified (general image datasets)	Emotional encoding fused with dense visuals	Sentiment-aware media
FaceGemma (2023) [194]	Multimodal fine-tuning with attribute prompts	FaceAttrib (CelebA subset)	40 facial attributes for nuanced captions	Portrait indexing; multilingual aids
OPCap (2024) [195]	YOLO-tiny object detection + CLIP attribute predictor + Transformer	COCO; nocaps	Object-aware prompting to reduce hallucinations	Image search; smart albums
Dual-path EVC (2024) [196]	Dynamic emotion perception + adaptive decoder (ResNet/3D-ResNeXt)	EVC-MSVD; EVC-VE; SentiCap	Emotion evolution modules for balanced captions	Video ads; social media engagement
Motion-CNN (2021) [197]	Faster R-CNN object detection + motion-CNN + LSTM attention	MSR-VTT2016-Image; MSCOCO	Motion features from object regions	Aiding visually impaired; indexing
BLIP-2-based (2023) [198]	BLIP multimodal fusion + object detection + FER cues	Surveillance datasets (custom complex scenes)	Vision–text integration for scene understanding	Fault monitoring in surveillance/Industry 4.0
FocusCap (2023) [199]	CLIP embeddings + pre-trained LM + guided FER attention	COCO; Visual Genome	Unsupervised object-focused captioning with emotional guidance	Real-time industrial diagnostics; accessibility

**Table 7:** Performance trade-offs of integrated approaches.

Model	Accuracy Gains (e.g., BLEU-4/CIDEr)	Computational Cost (GFLOPs)	Suitability for Industry 4.0 (e.g., Real-Time)
Face-Cap (2019) [112]	+5–10% emotional metrics	Low (∼10)	Moderate; adaptable for operator monitoring
FaceGemma (2023) [194]	METEOR +15% on attributes	Medium (∼50)	Low; high for offline analysis
OPCap (2024) [195]	Hallucination reduction 15–20%	High (∼80)	High; edge devices for fault detection
Dual-path (2024) [196]	CIDEr +10–15%	Medium (∼60)	Moderate; dynamic for PHM alerts
BLIP-2 (2023) [198]	SPICE +12–18% in complex scenes	High (∼70)	Moderate; suitable for surveillance-based fault awareness
FocusCap (2023) [199]	METEOR +10–15% (object-focused)	Medium (∼55)	High; zero-shot for industrial real-time

## Data Availability

All data analyzed in this review are from previously published studies, which are cited throughout the manuscript.
